# Machine learning assessment of vildagliptin and linagliptin effectiveness in type 2 diabetes: Predictors of glycemic control

**DOI:** 10.1371/journal.pone.0309365

**Published:** 2024-08-26

**Authors:** Hanin A. Esaifan, Loai M. Saadah, Khawla Abu Hammour, Rana K. Abu Farha

**Affiliations:** 1 Department of Clinical Pharmacy and Therapeutics, Faculty of Pharmacy, Applied Science Private University, Amman, Jordan; 2 Department of Biopharmaceutics and Clinical Pharmacy, Faculty of Pharmacy, The University of Jordan, Amman, Jordan; Faculty of Pharmacy, Ain Shams University, EGYPT

## Abstract

**Objective:**

Differential effects of linagliptin and vildagliptin may help us personalize treatment for Type 2 Diabetes Mellitus (T2DM). The current study compares the effect of these drugs on glycated hemoglobin (HbA1c) in an artificial neural network (ANN) model.

**Methods:**

Patients with T2DM who received either vildagliptin or linagliptin, with predefined exclusion criteria, qualified for the study. Two input variable datasets were constructed: with or without imputation for missing values. The primary outcome was HbA1c readings between 3 to 12 months or the reduction in HbA1c levels.

**Results:**

The cohort comprised 191 individuals (92 vildagliptin and 99 linagliptin). Linagliptin group had significantly higher disease burden. For imputed dataset, HbA1c was lower with linagliptin at 3 to 12 months (7.442 ± 0.408 vs. 7.626 ± 0.408, P < 0.001). However, there was a small yet significant difference in HbA1c reduction favoring vildagliptin over linagliptin (-1.123 ± 0.033 vs. -1.111 ± 0.043, P < 0.001). LDL level, uric acid, and the drug group were identified as predictors for HbA1c levels. In the non-imputed dataset HbA1c at 3 to 12 months was lower with linagliptin (median ± IQR: 7.489 ± 0.467 vs. 7.634 ± 0.467, P-value < 0.001). However, both linagliptin and vildagliptin exhibited similar reductions in HbA1c levels (both median ± IQR of -1.07 ± 0.02). Predictors for HbA1c levels included eGFR level and the drug group.

**Conclusion:**

Linagliptin effectively lowers HbA1c levels more than vildagliptin including in patients with comorbidities. DPP4-I choice is a constant predictor of HbA1c in all models.

## Introduction

Diabetes mellitus (DM) stands as a complex interplay of genetic predisposition, metabolic dysfunction, and autoimmune processes, escalating globally with an alarming prevalence [1]. The 10th edition of the International Diabetes Federation Atlas paints a stark picture. Approximately 537 million individuals aged 20–79 grapples with this metabolic disorder, emphasizing an urgent need for effective interventions [2]. At the heart of DM lies the peril of hyperglycemia, compounded by dyslipidemia, laying the groundwork for a spectrum of complications, from coronary artery diseases to microvascular impairments [3]. Central to mitigating these risks is the pursuit of glycemic control. The benchmark here is HbA1c levels below 7% as endorsed by leading diabetes associations. The pharmacotherapeutic landscape for Type 2 Diabetes Mellitus (T2DM) has witnessed a proliferation of options. The United States Food and Drug Administration (FDA) approved medications across diverse classes [4–6]. From traditional agents like sulfonylureas to newer iterations such as dipeptidyl peptidase 4 (DPP-4) inhibitors and glucagon-like peptide-1 (GLP-1) receptor agonists, the armamentarium has expanded. As a result, this expansion heralds a shift towards personalized treatment paradigms [7, 8]. Incretin-based therapies, typified by GLP-1 agonists and DPP-4 inhibitors, have emerged as stalwarts in T2DM management. They offer multifaceted benefits including enhanced insulin secretion and glucagon suppression. Among these, linagliptin shines for its unique profile, accommodating individuals with advanced renal impairment, underscoring the importance of tailored approaches in diabetes care [9–11].

However, comparing linagliptin to other DPP-4 inhibitors is impaired by many factors. For example, linagliptin, vildagliptin, sitagliptin, and saxagliptin had similar effectiveness when added to metformin and they were all superior to alogliptin [12]. However, this meta-analysis was based on randomized trials of individual agents that had different confounders by indication. One study showed that vildagliptin was superior to both sitagliptin and linagliptin in the reduction of HbA1c [13]. However, the reported fasting plasma sugar (FBS) was surprisingly lower in the linagliptin group and the duration of that study was 12 weeks post treatment. In reality, most patients need about 6 to 12 months to reach their nadir HbA1c reduction on incretin-based therapy [14, 15]. Another contradictory piece of evidence, to the one study above favoring vildagliptin, has found that linagliptin has the most potent DPP-4 inhibition in both mice and humans. In this study the equipotent 50% Inhibitory Concentration (IC50) of linagliptin and vildagliptin were 0.14 nmol/L (range 0.13–0.14) and 34 nmol/L (30.7–37.4), respectively [16]. The ratio of these equipotent doses is much larger than that of the actual therapeutic doses for the two agents in human subjects. A most recent double blinded, randomized, actively-controlled study found that adding linagliptin 5 mg to dapagliflozin 10 mg, both once daily, was superior to adding vildagliptin SR 100 mg once daily in all outcomes [17]. These outcomes included a greater reduction of HbA1c, FBS, postprandial blood glucose, and weight. Moreover, there was a greater proportion of patients achieving an HbA1c below 7% in the linagliptin group at 16 weeks post-baseline (all comparisons were statistically significant). However, up to this date, there is no direct head-to-head comparison of linagliptin and vildagliptin at nadir effectiveness in real world patients. Moreover, there is little utilization of artificial intelligence modeling to elucidate differential effects in practice that could support a personalized approach to prescribing DPP-4 inhibitors. These differential effects have to be further explored especially in light of anecdotal evidence that the safety of these drugs is also different. For example, patients with idiosyncratic liver injury can be successfully switched from vildagliptin to linagliptin [18]. Additionally, data has demonstrated differential effects for linagliptin and vildagliptin on the thyroid gland which is also an evolving topic in this area of research [19]. Machine learning can detect hidden relations of variables without making any statistical assumptions, like the ones made with multivariate analyses, using data with or without imputation. It also helps us determine the specific impact of personalized DPP4-I regimens on HbA1c reduction and levels.

Recent advancements in technology have revolutionized the way we approach complex datasets, particularly in the realm of healthcare. Machine learning, in particular, has emerged as a powerful tool capable of processing vast amounts of data beyond the capacity of the human brain. ANN, inspired by the functioning of the human brain, have proven instrumental in analyzing expansive bodies of research and clinical data. By extracting valuable insights, these technologies pave the way for new hypotheses and deeper understanding of medical conditions, including T2DM [20]. User-friendly software applications have facilitated the utilization of machine learning techniques in healthcare for both drug discovery as well as rational use of pharmacotherapy and clinical services [21–23]. These tools streamline the process of analyzing complex clinical data, enabling researchers to tackle challenging clinical problems with greater efficiency. The impact of such technological advancements is evidenced by recent studies. For example, embracing clinical pharmacy interventions, often overlooked by physicians, led to shorter hospital stays for patients in acute care settings [22]. Empowering clinical pharmacists to prescribe medications in critical care settings resulted in reduced lengths of intensive care stay as well. Additionally, administering palivizumab to specific subsets of neonatal intensive care unit infants during respiratory syncytial virus outbreaks significantly reduced the duration of supplemental oxygen use [23]. These findings not only influence the development of new guidelines but also drive progress across various fields of study [24, 25]. In this article, we aim to leverage ANN models to compare and contrast the effectiveness of two commonly used medications, vildagliptin and linagliptin, in managing T2DM. Additionally, we aim to assess the predictors influencing HbA1c levels. Our primary focus will be on HbA1c reported within 3 to 12 months of follow-up. These differences in vildagliptin and linagliptin effects on HbA1c reduction, if any, would help personalize treatments for patients.

## Subjects and methods

### Study design

This retrospective observational study conducted at Jordan University Hospital (JUH) involved adult patients diagnosed with T2DM, aged 18 years or older. Individuals were included if they commenced treatment with either linagliptin or vildagliptin between May 2018 and July 2021. The reason for these dates is that fact that linagliptin entered into the Jordan University Hospital formulary system in May 2018. To have an enough sample we needed to go to the full duration before the novel coronavirus disease of 2019 (COVID-19) pandemic actually started in Jordan in July 2020. We needed a follow-up of 1 year which is why we had the data taken till July 2021. Now all these patients were taken from the to outpatient record systems. Some of these patients may have been admitted during this period, However, we were able to see all their information and none were actually COVID-19 cases or received COVID-19 medications. The possibility that some may have caught the severe acute respiratory syndrome coronavirus -2 (SARS CoV-2) as outpatients can NOT be excluded. However, the fact that this is an acute and very likely mild case make us rest sure that COVID-19 did not affect HbA1c in our study. The study adhered to the guidelines outlined in the World Medical Association Declaration of Helsinki [26]. More details about ethics approval are provide in the ethics statement section below.

### Study subjects: Inclusion and exclusion criteria

Out-patients eligible for inclusion in this study were those aged 18 years or older with a confirmed diagnosis of T2DM. They were required to have received at least 9 documented dispensing of the index drug, indicating an adherence rate of 75% or higher, during the one-year follow-up period. Patients with type 1 diabetes mellitus (T1DM), diabetic ketoacidosis, prior treatment with other medications, those who discontinued or changed the index drug during the study period, and those lacking baseline HbA1c measurements were excluded. Additionally, patients without HbA1c values recorded between 3 to 12 months of follow-up were also excluded. Follow-up assessments were conducted at 3, 6, 9, and 12 months after initiating the index drug.

### Data collection

Information regarding prescribed linagliptin or vildagliptin, including initial prescription dates and total prescriptions, was obtained from JUH’s electronic system. Patient-specific data and prescribed medications were collected from medical records. Data access for research purposes began on July 12, 2023 and continued until November 25, 2023.

Clinical notes from specialized clinics were carefully reviewed to document baseline complications of T2DM and any associated comorbidities. The Age-adjusted Charlson Comorbidity Index (CCI), aligned with ICD-10 codes, was utilized for as an indicator of disease burden and comorbidities [27].

A pre-designed data collection sheet was systematically used to gather essential information from patients, ensuring comprehensive and standardized data collection.

### Data analysis

The data from the hospital database were analyzed to identify the total number of patients who initiated either vildagliptin or linagliptin based on their first pharmacy claim date. We applied inclusion and exclusion criteria to define the final study population. To ensure comparable sample size, we used randomization table generated by SPSS to randomly select patients in case of different sizes in the groups.

To identify relevant input variables, we employed SPSS univariate comparison of baseline characteristics, retaining unmatched variables meeting a significance level of 0.05. Additionally, we performed multiple imputation of missing values using the expectation maximization (MIEM) procedure, recognized as one of the most robust methods even with high missingness ratios [28]. Littles’ Missing Completely at Random (MCAR) test was utilized to assess missing data patterns, indicating that missingness was MCAR [29]. Imputed data were evaluated by model fit checks and univariate comparisons were conducted again for imputed datasets regarding baseline characteristics.

Subsequently, the selected unmatched input variables were incorporated into the development of the ANN model. This involved building, training, testing, and cross-validating the ANN model to explore its predictive capabilities for various outcomes.

These data analysis methods have been previously used [21–23]. The rationale is that our methods ensure selecting the largest number possible of input variables that may influence the primary outcome. Univariate analyses then help us minimize the actual inputs used in the ANN model and thus overcome the problems of insufficient sample sizes and overtraining while at the same time providing robust insights on the effect of the remaining variables on the final outcome.

### Definitions

Definitions related to ANN modeling were previously published [21–23]. Variable impact is a calculated percentage that represent the amount of variability explained by a given input variable in the ANN model. The larger this value, the more important the variable.

### Study primary endpoint

Our primary outcome was the first reported HbA1c level following the initiation of treatment with either drug at 3 to 12 months follow-up. In another model primary outcome was HbA1c reduction from baseline. Several interim analyses were done with data available at different times during the study. Reassignment analyses were implemented to compare secondary endpoints of HbA1c with varying levels of eGFR and uric acid.

### Model development

Based on the SPSS univariate comparison of baseline characteristics considering imputed and non-imputed datasets “S1 Table”, mismatched variables were identified. To streamline our analysis for the ANN model development, we organized these variables into domains. This categorization aimed to eliminate any redundant emphasis on specific aspects or diseases among the variables. The purpose was to optimize our sample size for the ANN, ensuring sufficiency for the included variables yet simplifying the process of managing and incorporating them into our model. Our expert estimation of the sample size needed for ANN training and testing was calculated as follows:

Samplesize=20NumberofInputVariables (1)

Our primary endpoint was the initial HbA1c measurement following the commencement of treatment with each drug. Typically, these measurements were taken at specified intervals: at 3, 6, 9, and 12 months. All the patients had at least 1 value during follow-up from 3 to 12 months.

### Data sensitivity analysis

Subsequently, a sensitivity analysis was conducted to identify the optimal percentage of data for actual training, with the remaining portion allocated for internal validation, the ANN sensitivity analysis showed that the best distribution of training and testing would be 85%/15%. Therefore, the sample size of 20 x input variables will be divided into 85% for training and 15% for internal validation (testing).

### Data training

The training settings were configured with default parameters, except for a few specific adjustments. Live predictions were enabled, and the predicted values were directly placed into target cells. Additionally, variable impacts were automatically calculated after each training session. The Best Net search option was utilized with the number of nodes in both hidden layers automatically selected through the Multi-Layer Feed Forward Network (MLFN) method. Furthermore, all network configurations tested during the process were saved. To ensure comprehensive training, a variety of stop conditions were implemented, such as training for more than 2 hours, exceeding 1 million trials, or observing less than a 1% change in error (or no change in error) within the last hour of training.

### Statistical analysis

The Statistical Package for Social Sciences (SPSS) version 26 was used to code, input, and analyze the obtained data (SPSS Inc., Chicago, IL, USA). For continuous variables, the median and Interquartile range (IQR) were used, and for categorical variables, frequencies and percentages were used. The Shapiro-Wilk test was used to determine if a continuous variable is normally distributed (P> 0.05). To assess inter-group variations, the chi-square test and Mann-Whitney U test were applied. In terms of paired comparisons of continuous variables, we used the Wilcoxon signed-rank test. Significance was established at a threshold of p ≤ 0.05. All tests were two-tailed.

### Ethics statement

This study was approved by the Institutional Review Board (IRB) of [Jordan University Hospital] (protocol number [10 2023/16436]), approval date July 12, 2023 and for 1 year, (renewable). The study was conducted in accordance with the ethical standards laid down in the 1964 Declaration of Helsinki and its later amendments. Confidentiality and anonymity of participants were ensured throughout the research process.

## Results

**Fig 1:**
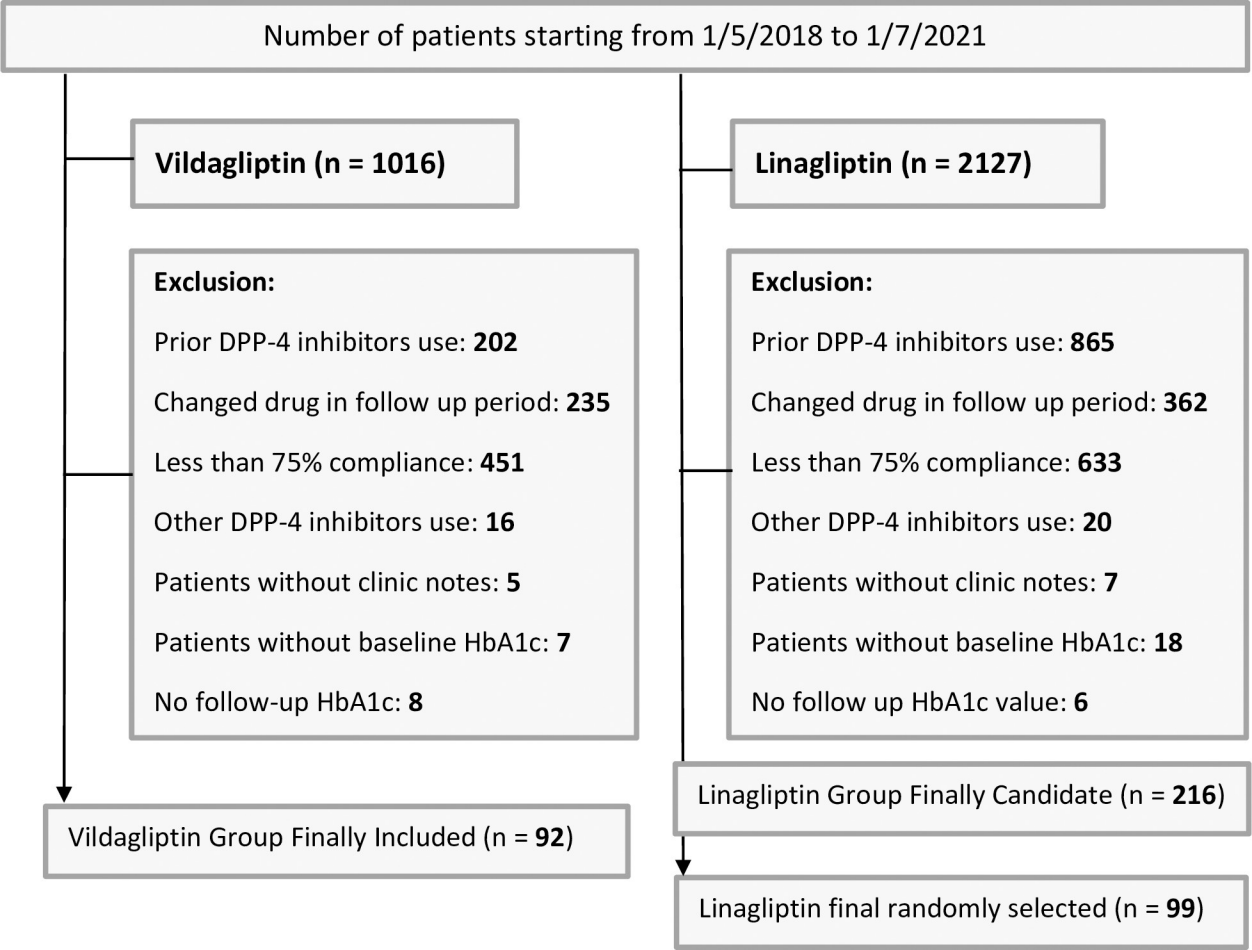
Data collection, inclusion, exclusion, and analysis. Flow diagram of vildagliptin and linagliptin patient recruitment process.

The process of patient inclusion and refinement is depicted in “**Fig 1”**. Initially, 1,016 patients who initiated vildagliptin were identified. After the exclusion criteria were applied, a final cohort of 92 patients was included for analysis. For linagliptin, a total of 2,127 patients were identified within the specified period. To ensure a comparable sample size to that of the vildagliptin group, approximately 45% of the 216 linagliptin patients who meet inclusion/exclusion criteria were randomly selected using a randomization table generated by SPSS. This randomization process and applying inclusion/exclusion criteria resulted in 99 linagliptin patients being recruited and included in the final analysis.

Now, 18 variables were mismatched for the imputed dataset “Table 1” and 12 without imputations “Table 2”. To streamline our analysis for the ANN model development, we organized these variables into domains. This categorization aimed to eliminate any redundant emphasis on specific aspects or diseases among the variables. The purpose was to optimize our sample size for the ANN, ensuring sufficiency for the included variables yet simplifying the process of managing and incorporating them into our model.

**Table 1:** Baseline characteristics of enrolled patients–imputed database (n = 191). Median ± Interquartile range (IQR) for continuous data and number (percentage) for categorical variables.

Parameter (Variable)	Domain	Vildagliptin (n = 92)	Linagliptin (n– 99)	P-value
Age (years)	Demographic	58.6 ± 12.3	63 ± 16.5	0.002*
Weight (Kg)	Demographic	90 ± 21	83 ± 23.3	0.369
Female	Demographic	40 (43.5)	48 (48.5)	0.488
Duration of DM (years)	Diabetes	6 ± 8	7 ± 8	0.983
CCI	Demographic	3 ± 3	4 ± 3	0.003*
Dyslipidemia	Dyslipidemia	88 (95.7)	95 (96)	1.000
Hypertension	Cardiovascular	69 (75)	89 (89.9)	0.006*
Hypothyroidism	Thyroid	9 (9.8)	21 (21.2)	0.030*
Bone and joint disease	Miscellaneous	16 (17.4)	26 (26.3)	0.139
Diabetic neuropathy	Diabetes	6 (6.5)	12 (12.1)	0.186
Diabetic nephropathy	Renal	1 (1.1)	19 (19.2)	0.001*
Diabetic retinopathy	Diabetes	11 (12)	14 (14.1)	0.655
Concomitant OHA		#N/A	#N/A	
• Metformin	Diabetes	84 (91.3)	82 (82.8)	0.083
• Insulin	Diabetes	23 (25)	34 (34.3)	0.159
• Sulfonylurea	Diabetes	47 (51.1)	50 (50.5)	0.936
Dyslipidemia drug	Dyslipidemia	86 (93.5)	95 (96)	0.526
Antihypertensive drug	Cardiovascular	64 (69.6)	82 (82.8)	0.031*
Cardiovascular drug**	Cardiovascular	50 (54.3)	68 (68.7)	0.042*
HbA1c (%)	Diabetes	8.3 ± 2.3	7.9 ± 2.3	0.195
T-C (mg/dL)	Dyslipidemia	167.5 ± 36.5	153 ± 46	0.002*
LDL-C (mg/dL)	Dyslipidemia	111.7 ± 38.4	88.3 ± 44.2	0.001*
HDL-C (mg/dL)	Dyslipidemia	40.5 ± 12	42 ± 12	0.810
TG (mg/dL)	Dyslipidemia	175 ± 77.8	183 ± 67.5	0.323
AST (U/L)	Liver	18.6 ± 3.3	20.2 ± 3.5	0.001*
ALT (U/L)	Liver	22.3 ± 6.8	25.4 ± 9.6	0.065
ALP (U/L)	Liver	110.2 ± 0	92.2 ± 18	0.001*
Calcium (mg/dL)	Miscellaneous	9.7 ± 0.1	9.5 ± 0.5	0.001*
Uric acid (mg/d)	Miscellaneous	5.4 ± 0	6.6 ± 0	0.001*
eGFR (ml/min/1.73 m2)	Renal	105 ± 39.8	83 ± 52	0.001*
Serum Cr (mg/dL)	Renal	0.7 ± 0.3	0.8 ± 0.6	0.001*
Blood Urea (mg/dL)	Renal	30.5 ± 8.9	36.6 ± 19.2	0.001*
Urine Albumin (mg/g)	Renal	2.3 ± 0	1.3 ± 0	0.001*
Urine Cr (mg/dL)	Renal	1.9 ± 0.4	1.3 ± 0.3	0.001*

* Significant at 0.05 significance level. To assess inter-group variations, the chi-square test and Mann-Whitney U test were applied.

**Cardiovascular medications include thrombolytic, anti-platelets, oral anticoagulants

**Abbreviations**: **AST**: Aspartate transaminase; **ALT**: Alanine aminotransferase; **CCI**: Charlson Comorbidity Index Score. **Cr**: Creatinine; **eGFR**: estimated glomerular filtration rate; **HbA1c**: Hemoglobin A1c; **HDL-C**: High-density lipoprotein cholesterol; **LDL-C**: Low-density lipoprotein cholesterol; **OHA**: Oral hypoglycemic agent; **T-C**: Total cholesterol; **TG**: Triglyceride.

**Table 2:** Baseline characteristics of enrolled patients–non-imputed database (n = 191). Median ± IQR for continuous data and number (percentage) for categorical variables.

Parameter (Variable)	Domain	Vildagliptin (n = 92)	Linagliptin (n– 99)	P-value
Age (years)	Demographic	58.6 ± 12.3	63 ± 16.5	0.002*
Weight (Kg)	Demographic	90 ± 21	83 ± 23.3	0.369
Female	Demographic	40 (43.5)	48 (48.5)	0.488
Duration of DM (years)	Diabetes	6 ± 8	7 ± 8	0.983
CCI	Demographic	3 ± 3	4 ± 3	0.003*
Dyslipidemia	Dyslipidemia	88 (95.7)	95 (96)	1.000
Hypertension	Cardiovascular	69 (75)	89 (89.9)	0.006*
Hypothyroidism	Thyroid	9 (9.8)	21 (21.2)	0.030*
Bone and joint disease	Miscellaneous	16 (17.4)	26 (26.3)	0.139
Diabetic neuropathy	Diabetes	6 (6.5)	12 (12.1)	0.186
Diabetic nephropathy	Renal	1 (1.1)	19 (19.2)	0.001*
Diabetic retinopathy	Diabetes	11 (12)	14 (14.1)	0.655
Concomitant OHA				
• Metformin	Diabetes	84 (91.3)	82 (82.8)	0.083
• Insulin	Diabetes	23 (25)	34 (34.3)	0.159
• Sulfonylurea	Diabetes	47 (51.1)	50 (50.5)	0.936
Dyslipidemia drug	Dyslipidemia	86 (93.5)	95 (96)	0.526
Antihypertensive drug	Cardiovascular	64 (69.6)	82 (82.8)	0.031*
Cardiovascular drug**	Cardiovascular	50 (54.3)	68 (68.7)	0.042*
HbA1c (%)	Diabetes	8.3 ± 2.3	7.9 ± 2.3	0.195
T-C (mg/dL)	Dyslipidemia	162.3 ± 47.3	147.2 ± 65	0.06
LDL-C (mg/dL)	Dyslipidemia	107.5 ± 51	82 ± 55.5	0.008*
HDL-C (mg/dL)	Dyslipidemia	40 ± 14	42 ± 14	0.875
TG (mg/dL)	Dyslipidemia	154 ± 88.2	176.2 ± 115.8	0.474
AST (U/L)	Liver	16 ± 10.1	18.7 ± 9.7	0.288
ALT (U/L)	Liver	19.1 ± 16.1	21.5 ± 11.2	0.56
ALP (U/L)	Liver	89 ± 31.3	95 ± 32.5	0.826
Calcium (mg/dL)	Miscellaneous	9.6 ± 0.7	9.4 ± 0.6	0.413
Uric acid (mg/dL)	Miscellaneous	5.4 ± 1.7	6.6 ± 2	0.050*
eGFR (ml/min/1.73 m2)	Renal	103.8 ± 46.1	84.3 ± 61.9	0.001*
Serum Cr (mg/dL)	Renal	0.7 ± 0.3	0.8 ± 0.6	0.001*
Blood Urea (mg/dL)	Renal	30 ± 13.1	37.4 ± 26.4	0.002*
Urine Albumin (mg/g)	Renal	0 ± 1.5	0 ± 1	0.684
Urine Cr (mg/dL)	Renal	1.4 ± 1.2	1.2 ± 1	0.471

* Significant at 0.05 significance level. To assess inter-group variations, the chi-square test and Mann-Whitney U test were applied.

**Cardiovascular medications include thrombolytic, anti-platelets, oral anticoagulants

**Abbreviations**: **AST**: Aspartate transaminase; **ALT**: Alanine aminotransferase; **CCI**: Charlson Comorbidity Index Score. **Cr**: Creatinine; **eGFR**: estimated glomerular filtration rate; **HbA1c**: Hemoglobin A1c; **HDL-C**: High-density lipoprotein cholesterol; **LDL-C**: Low-density lipoprotein cholesterol; **OHA**: Oral hypoglycemic agent; **T-C**: Total cholesterol; **TG**: Triglyceride.

We conducted an assessment of the potential relationship between variables and HbA1c, aiming to select the most representative variable within different domains. First with **no imputations,** CCI, largely influenced by age and not predictive of HbA1c, was excluded from our ANN model. Age and all drug variables, irrespective of their indications, were included in the inputs, along with variables for hypertension, hypothyroidism, and LDL-C. Considering the limited sample size and the presence of four indicators of DPP4-I use related to kidney function, we chose to include one continuous (eGFR) and one categorical (diabetic nephropathy) of these. Thus, the final set of variables for our ANN model comprised 8 individual variables and the DPP4-I group, totaling 9 variables. **For the imputed data sets**, we tried to include new unmatched variables as we can and also by using representative factors from each domain. We had the following inputs included in the ANN model for the imputed dataset; DPP4-I groups, hypertension, hypothyroidism, age, baseline LDL-C, calcium, uric acid, urine albumin and eGFR.

This means we can train the ANN model on 20 x 9 = 180 cases and use the remaining 11 cases for external validation. These 11 cases were selected randomly from the total of 191 cases. Notably, the linagliptin group exhibited a higher burden of illness, characterized by advanced age, hypertension, hypothyroidism, and renal disease. This places the linagliptin group at a disadvantage in terms of reducing their HbA1c levels. Conversely, the vildagliptin group started with higher baseline HbA1c levels although non-significant, providing them with a potential advantage in responding to DPP4-I treatment. Furthermore, the greater and lower percentage of vildagliptin patients on metformin and insulin therapy at baseline and subsequent time points, respectively, although not statistically significant, increases the likelihood of observing HbA1c reduction in the vildagliptin group and indicate a higher disease burden once again in the linagliptin group. Finally, a higher CCI indicated a higher disease burden in the linagliptin group as well.

Our primary endpoint was the initial HbA1c measurement following the commencement of treatment with each drug. Typically, these measurements were taken at specified intervals: at 3 months for around 53 patients using Vildagliptin and 67 patients using Linagliptin, at 6 months involving 24 patients using Vildagliptin and 21 patients using Linagliptin, at 9 months for approximately 5 patients using Vildagliptin and 4 patients using Linagliptin, and at 12 months encompassing about 10 patients using Vildagliptin and 7 patients using Linagliptin.

### Artificial neural networks model development

**Fig 2:**
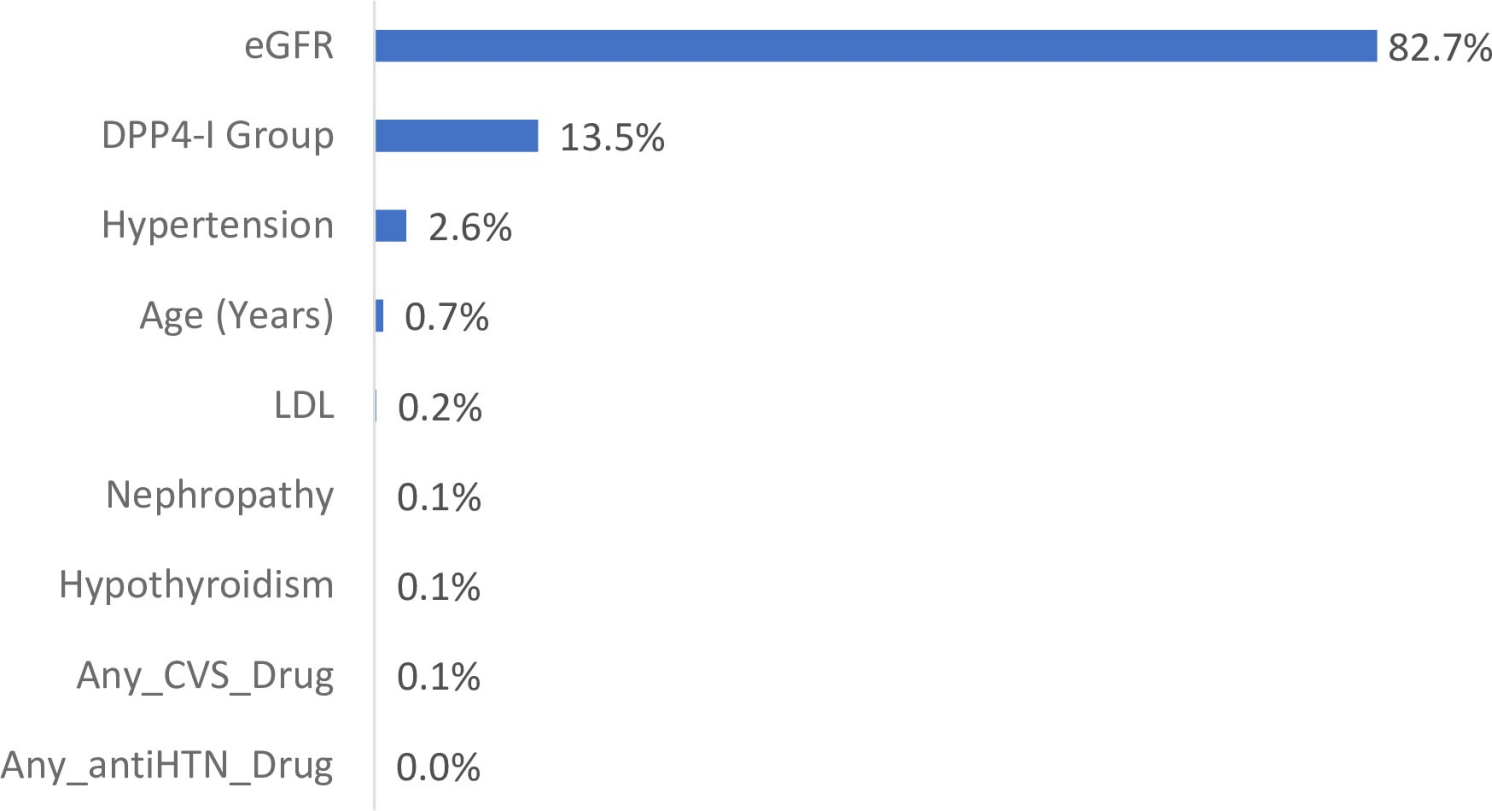
Variable impacts from best ANN model for the non-imputed dataset. GRNN and first reported HbA1c 3 to 12 month.

In the dataset **without imputations**, the first run focused on using the first recorded HbA1c reading post baseline as the primary outcome. After the completion of the Best Net search, linear predictor was the best configuration for the data (lowest RMS error of 1.81), general regression neural network (GRNN) was a close second best (1.82). When we used HbA1c reduction as the primary outcome in a second run, GRNN emerged as the best net configuration. In terms of external validation, this was suboptimal as expected, with low correlation factor (R^2^ = 0.026). Variable impacts are presented in “**Fig 2”.** These show that DPP4-I group drug was the second most important variable, following only eGFR. Reassignment analyses showed lower first HbA1c reported at 3 to 12 months’ post baseline with linagliptin (median ± IQR: vildagliptin 7.634 ± 0.467 vs. linagliptin 7.489 ± 0.467, P-value < 0.001). However, in the second run, linagliptin and vildagliptin had similar HbA1c reduction (both median ± IQR of -1.07 ± 0.02). Bear in mind the greater baseline disease burden in linagliptin group. Now, we have looked at few other reassignments for the **non-imputed** models to study the results. If all patients eGFR is set to 60, linagliptin in the non-imputed model is associated with lower HbA1c levels (vildagliptin 7.346 ± 0.414 vs. linagliptin 7.202 ± 0.414, P value < 0.001). Also, if eGFR is set to 30, linagliptin was better than vildagliptin (vildagliptin 7.102 ± 0.414 vs. linagliptin 6.958 ± 0.414, P value < 0.001). If eGFR is set to 120 the results are again favorable for linagliptin (7.835 ± 0.414 vs. 7.691 ± 0.414, P value < 0.001).

**Fig 3:**
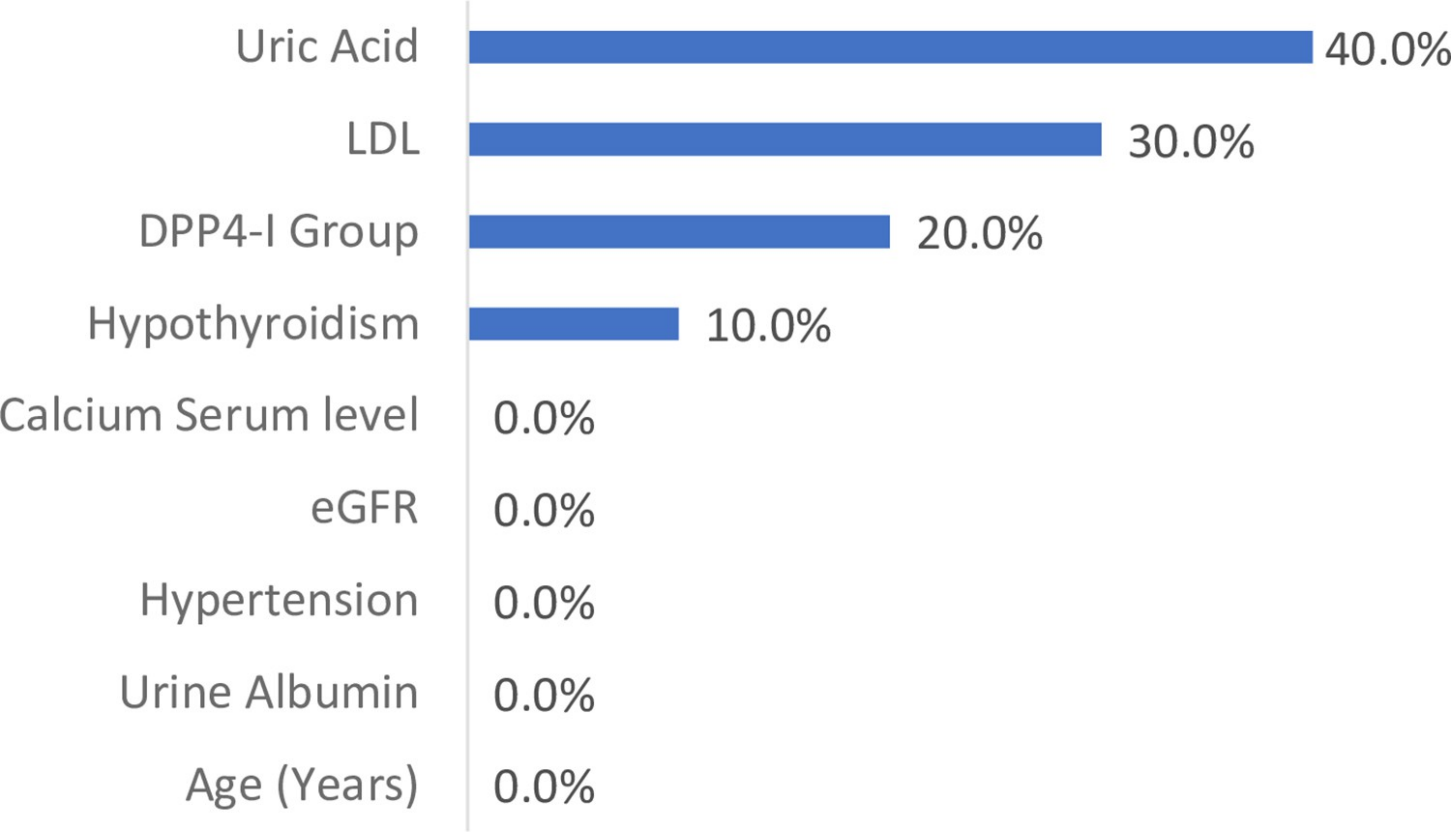
Variable impacts from best ANN model for the imputed dataset. GRNN and first reported HbA1c 3 to 12 month.

Now for the **imputed datasets**, the first HbA1c reported post baseline ANN converged in a similar way to the non-imputed dataset and showed the best net search results with linear predictor being the best model (RMS = 1.27). GRNN was again the second-best configuration (RMS = 1.36). |”**Fig 3”** shows the variable impacts for the GRNN model. Once again, the only robust variable was DPP4-I which was the third most important variable after Uric acid and LDL-C. For external validation results, it is clear that the linear predictor is suboptimal with low correlation factor (R^2^ = 0.181). Linagliptin was associated with a lower first reported HbA1c at 3 to 12 months’ post baseline (linagliptin 7.442 ± 0.408 vs. vildagliptin 7.626 ± 0.408, P < 0.001). For HbA1c reduction, there was a small yet significant reduction in HbA1c in vildagliptin versus linagliptin (Vildagliptin -1.123 ± 0.033 versus linagliptin -1.111 ± 0.043, P < 0.001). This is probably again largely influenced by the greater disease burden in linagliptin. Further, we explored these results with a few reassignments for the imputed models. Varying the uric acid from minimum value of 2.8 to maximum value of 10.6 showed that the lower HbA1c levels associated with linagliptin were still maintained. For uric acid level of 2.8 vildagliptin vs. linagliptin (7.799 ± 0.407 vs. 7.615 ± 0.407). For uric acid level of 10.6 vildagliptin vs. linagliptin (7.350 ± 0.407 vs. 7.166 ± 0.407). For LDL-C of 25 vildagliptin vs. linagliptin (7.744 ± 0.423 vs. 7.560 ± 0.423). For LDL-C of 228 vildagliptin vs. linagliptin (7.372 ± 0.423 vs. 7.188 ± 0.423). All P values were highly significant for these comparisons.

Study had a total of 12 runs and ANN models. Eight of these runs were interim on different data sets as the data arrived. Later more data were available and we were able to train and perform four final runs with two each on imputed and non-imputed datasets. One each of these two runs were on HbA1c level at 3 to 12 months and the other on HbA1c reduction. The mismatched variables in these were also different for these as clearly shown in “**Figs 2 and 3**”. Results in all runs and ANN models were consistent and demonstrated favorable effects for linagliptin in terms of HbA1c levels at 3 to 12 months and HbA1c reduction. Although, confounders may be present, the study findings were robust in all the ANN runs and models. Therefore, we strongly believe that these results can easily be reproduced by other researchers. Moreover, we have performed more runs later at individual time points where possible 3, 6, 9 and 12 months and the same findings manifested.

## Discussion

In this retrospective study involving 191 Jordanian patients, significant differences emerged between the vildagliptin and linagliptin cohorts, particularly in demographics and comorbidities. The linagliptin group skewed towards older age and had a higher prevalence of comorbid conditions like hypertension, hypothyroidism, and diabetic nephropathy. These factors contributed to a higher CCI score and increased use of antihypertensive medications in the linagliptin group. This trend suggests a clinical preference for prescribing linagliptin to patients with chronic kidney disease (CKD), a condition more common in older age and often associated with hypertension. Notably, several studies have reported a higher prevalence of hypothyroidism in individuals with advanced CKD compared to those without CKD [30–32].

In the investigation of linagliptin and vildagliptin as adjunct therapies for uncontrolled diabetic patients, all ANN models supported the efficacy of both agents in reducing HbA1c levels at 3 to 12 months. Previous studies have demonstrated a significant decline in HbA1c values; vildagliptin induced a reduction of 1.33%, compared to a 0.81% reduction observed with linagliptin after 3 months of treatment [13]. A comprehensive systematic review echoed these findings, concluding that vildagliptin achieved a 0.98% reduction in HbA1c, while a 0.60% reduction was attained with linagliptin [33]. On a contrasting note, linagliptin consistently exhibited noteworthy effectiveness in reducing HbA1c levels throughout the current study duration. Bearing in mind the unique challenges presented by older patients with comorbidities such as hypertension and hypothyroidism, this finding is crucial. Moreover, in this cohort, the presence of diabetic nephropathy may have necessitated the discontinuation of metformin and introduction of insulin but these also biased the findings to vildagliptin. These factors collectively suggest a scenario were achieving a substantial reduction in HbA1c becomes more challenging with any diabetic medication. However, linagliptin demonstrated its effectiveness in addressing these challenges. This effectiveness in elderly patients (>60 years old) was further corroborated by several studies highlighting the safety and efficacy of linagliptin in lowering HbA1c [34]. Furthermore, linagliptin demonstrated efficacy in lowering blood glucose levels in patients with a low baseline HbA1c of 7% after just one month of treatment [35]. The synergistic effect of linagliptin with metformin was also evident, showcasing its superior effectiveness when used in combination to control blood glucose levels compared to its alone use [36]. In line with these findings, another very recent study found that linagliptin added to dapagliflozin is in fact superior to vildagliptin in improving all the outcomes when compared in a double blind, randomized, controlled fashion [17]. The current real-world study further weighs on this finding and give more evidence that linagliptin is indeed a more effective DPP4-I regardless of the background diabetes pharmacotherapy.

In the assessment of variable impact on HbA1c levels, the drug group emerged as the second factor in the non-imputed model and the third in the imputed model affecting HbA1c levels. Many previous studies have highlighted the hypoglycemic agent as a crucial predictor of HbA1c levels, alongside factors like age, BMI, duration of diabetes mellitus (DM), baseline HbA1c, eGFR, total cholesterol, triglycerides, and HDL levels [37–40]. However, the current study shows that using ANN reveals that DPP4-I in these models is the only constant impactful variable in all runs. Therefore, this highlights the importance of personalized drug therapy in these patients. In the non-imputed dataset, linagliptin exhibited more effective reduction of HbA1c levels compared to vildagliptin across different estimated glomerular filtration rate (eGFR) levels. This finding aligns with numerous studies consistently reporting linagliptin’s efficacy in patients with varying degrees of renal impairment. The cumulative evidence suggests that linagliptin maintains a favorable reduction in HbA1c levels and a renal safety profile, even in individuals with compromised renal function [41, 42]. In contrast, in the imputed dataset, linagliptin demonstrated superior effectiveness in lowering HbA1c levels compared to vildagliptin across different uric acid levels. However, the relationship between uric acid and HbA1c levels remains controversial. Some studies suggest a negative association between serum uric acid and FBS and HbA1c levels [43]. Conversely, contrasting studies have shown significant elevations in serum uric acid, FBS, and HbA1c levels in patients with type 2 diabetes compared to healthy subjects [44]. Additionally, regression analyses have indicated an inverse association between serum uric acid levels and diabetes mellitus [45]. While others have yet shown worsening serum uric acid levels in poorly controlled type 2 diabetic subjects [46, 47]. Hence, further evaluation is needed to elucidate the relationship between uric acid levels and HbA1c levels. In the current study, nevertheless, linagliptin was again superior in reducing HbA1c levels across the board of uric acid reassignments and levels. And since all regression, ANN models, and external validation is quite poor in this area, it is advisable not to blindly follow imputed datasets for personalizing DPP4-I choices for diabetic patients.

### Limitations

The present study acknowledges several limitations that necessitate consideration. Notably, the research was conducted with a relatively small sample size at a single institution, potentially limiting the generalizability of the findings. However, the methods used overcome limited sample size problem and ensure robust findings from the ANN model. Therefore, we believe that another strength of this study is that the findings were consistent across all ANN runs and in all trained networks. Hence, well conducted studies will very likely concord with our findings.

### Future perspectives

Future investigations should consider extending the study duration to provide a more comprehensive understanding of the sustained efficacy and safety profiles of these medications. Furthermore, research must evaluate the efficacy and safety of linagliptin especially in patients without renal issues. Diversifying the study population across various demographic and comorbidity profiles is essential for enhancing the external validity of the results. Finally, incorporating real-world data analyses, including factors such as patient adherence and lifestyle considerations, would contribute valuable insights to the medications’ effectiveness and tolerability in routine clinical practice. These recommendations aim to guide future research endeavors, refining our understanding of vildagliptin and linagliptin in the context of diabetes management. However, with all that said, there seems a consistent and maintained superior effectiveness of linagliptin across a large board of ANN models and runs.

## Conclusion

Our retrospective study compared vildagliptin and linagliptin among Jordanian diabetes patients at a tertiary teaching hospital in Jordan. This study underscores linagliptin’s effectiveness in reducing HbA1c levels, particularly in elderly patients with comorbidities and varying renal function. Despite the challenges posed by age and comorbidities, linagliptin maintains its effectiveness, making it a promising option for managing uncontrolled diabetes in such populations. The study identified eGFR, uric acid, LDL level, and drug group as predictors for HbA1c. However, further research is warranted to clarify the relationship between uric acid levels and HbA1c reduction in diabetic patients. More exploration of the personalized pharmacotherapy choices is also much encouraged in diabetic patients.

## Supporting information

S1 Table Datasets for both the imputed and non-imputed data for ANN models. (XLSX)

## Abbreviation list

ANN: Artificial Neural Network
BMI: Body Mass Index
CCI: Charlson Comorbidity Index
CKD: Chronic Kidney Disease
COVID-19: novel Coronavirus Disease of 2019
DM: Diabetes Mellitus
DPP4-I: Dipeptidyl Peptidase 4 Inhibitor
FBS: Fasting Blood Sugar
FDA: Food and Drug Administration
eGFR: estimated Glomerular Filtration Rate
GLP-1: Glucagon-like peptide-1
GRNN: Generalized |Regression Neural Network
HbA1c: Glycated Hemoglobin
HDl: High Density Lipoprotein
IC50: Inhibitory Concentration 50%
ICD-10: International Classification of Diseases 10^th^ Revision
IQR: Interquartile Range
IRB: Institutional Review Board
JUH: Jordan University Hospital
LDL-C: Low Density Lipoprotein—Cholesterol
MCAR: Littles’ Missing Completely at Random test
MIEM: Multiple Imputation of missing values using the Expectation Maximization
MLFN: Multi-Layer Feedforward Network
OHA: Oral Hypoglycemic agent
RMS: Root Mean Square
SARS CoV-2: Severe Acute Respiratory Syndrome Coronavirus—2
SPSS: Statistical Package for Social Sciences software
SR: Sustained Release
T1DM: Type 1 Diabetes Mellitus
T2DM: Type 2 Diabetes Mellitus
VI: Variable Impact
